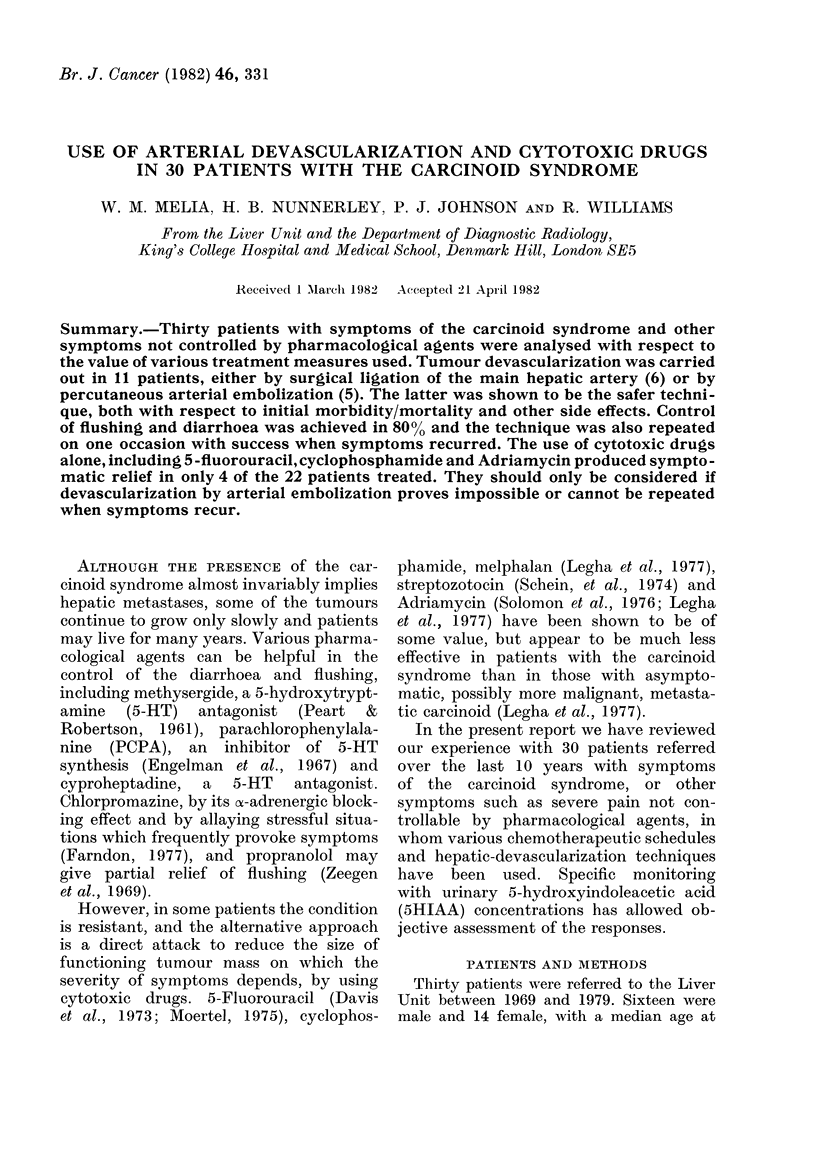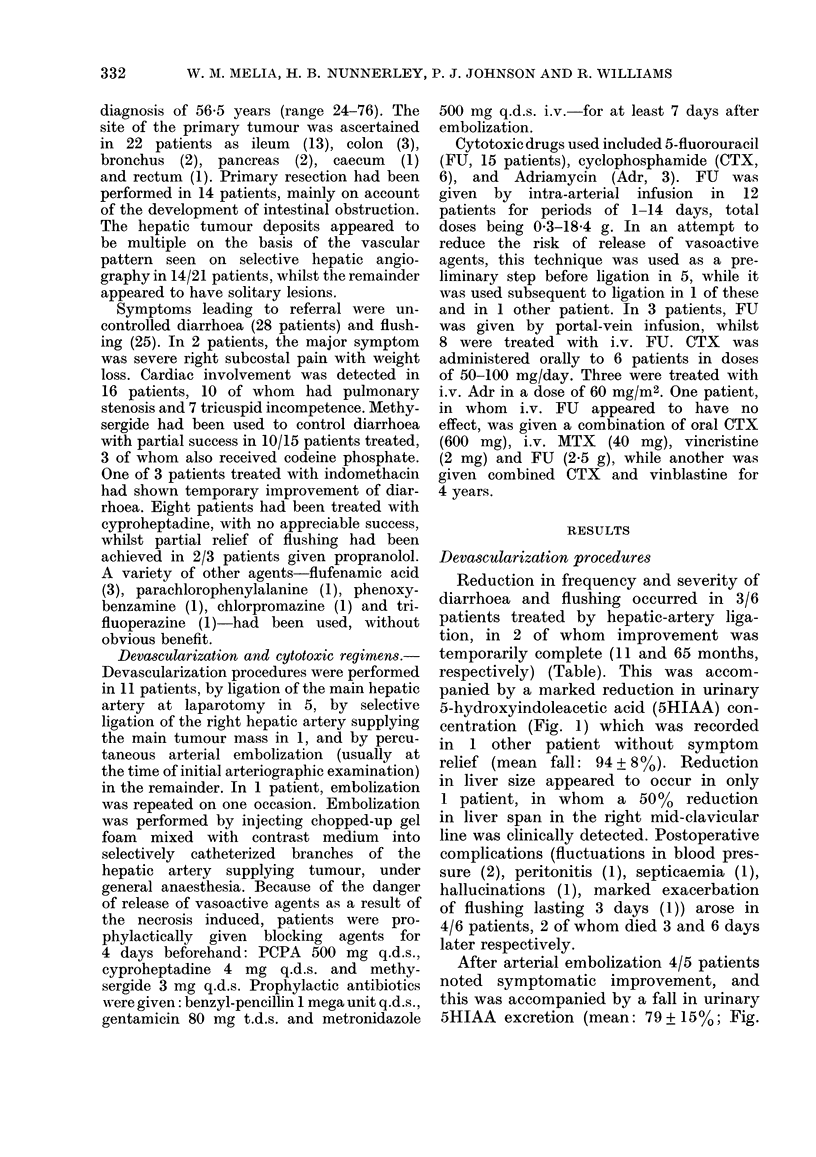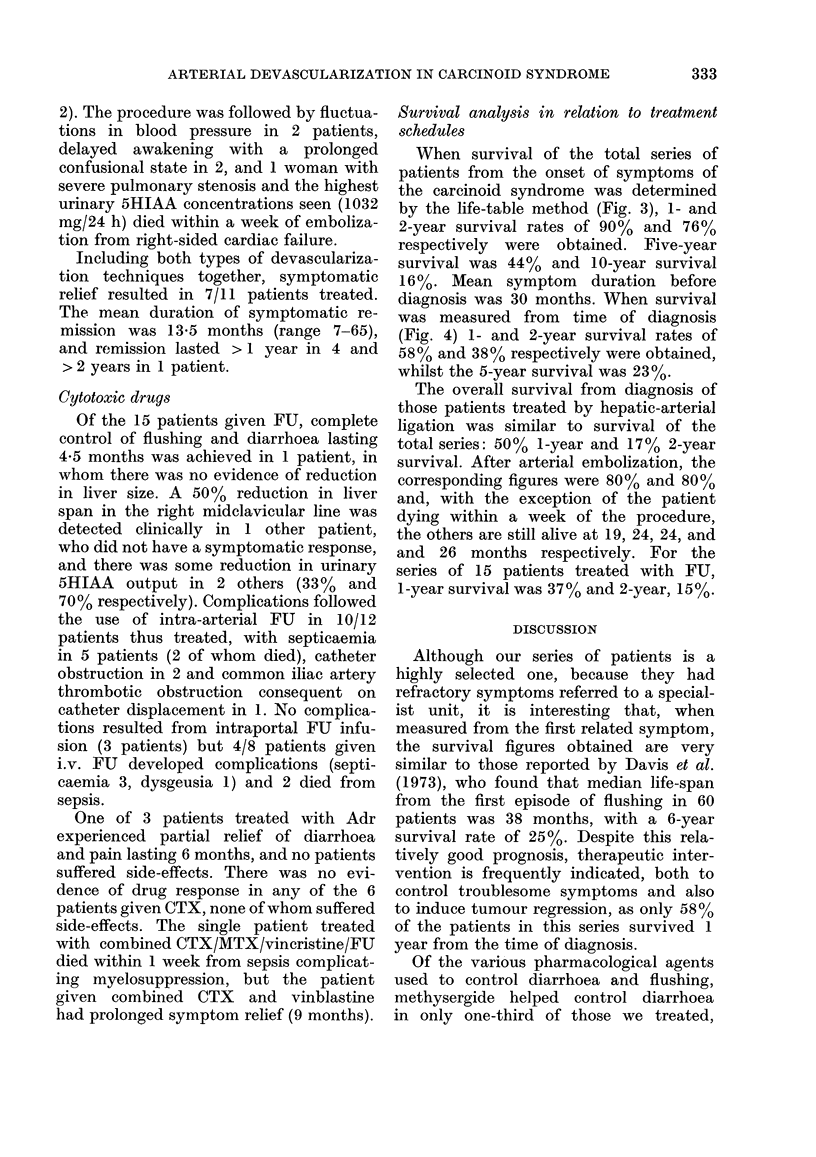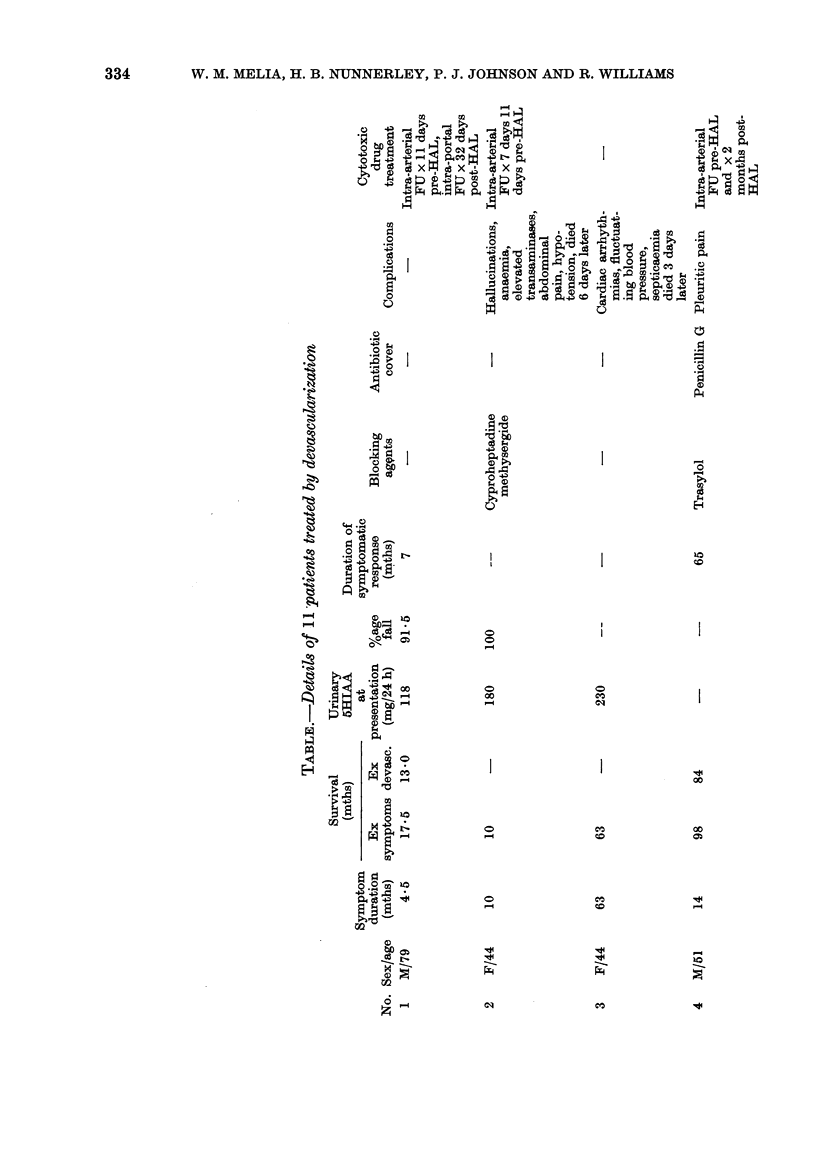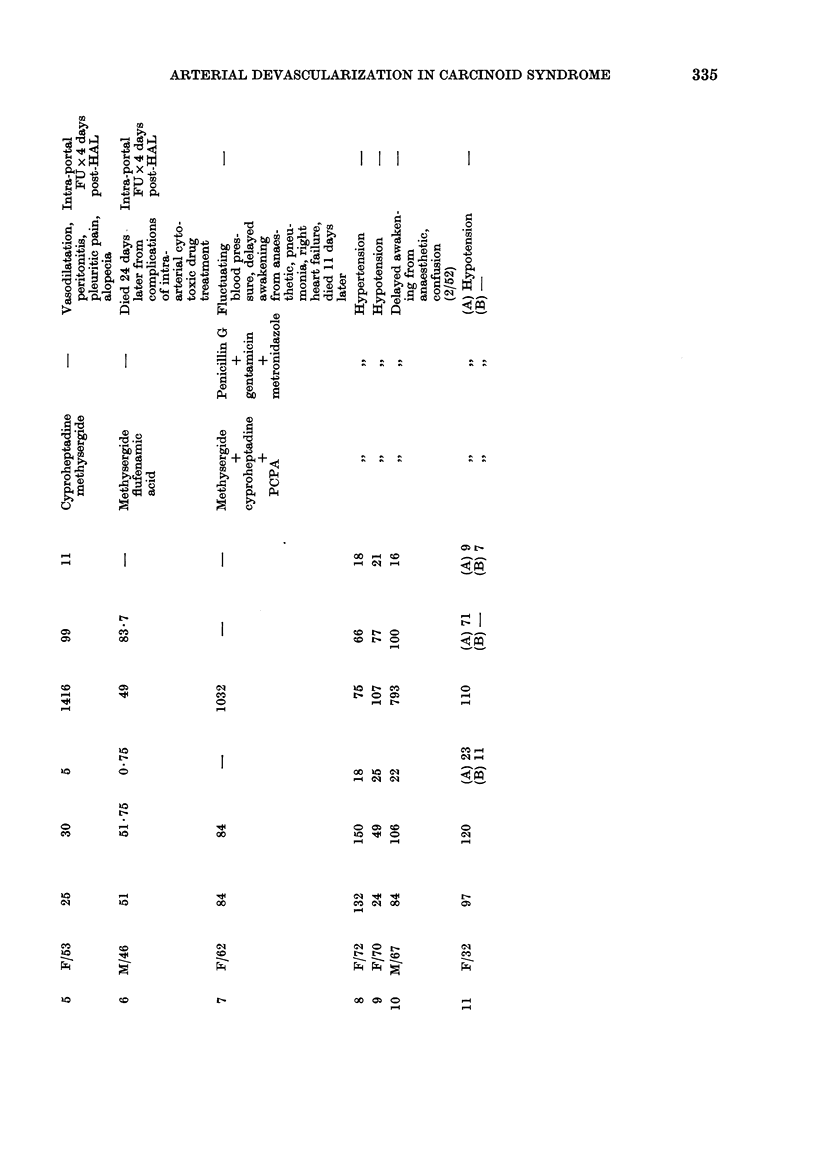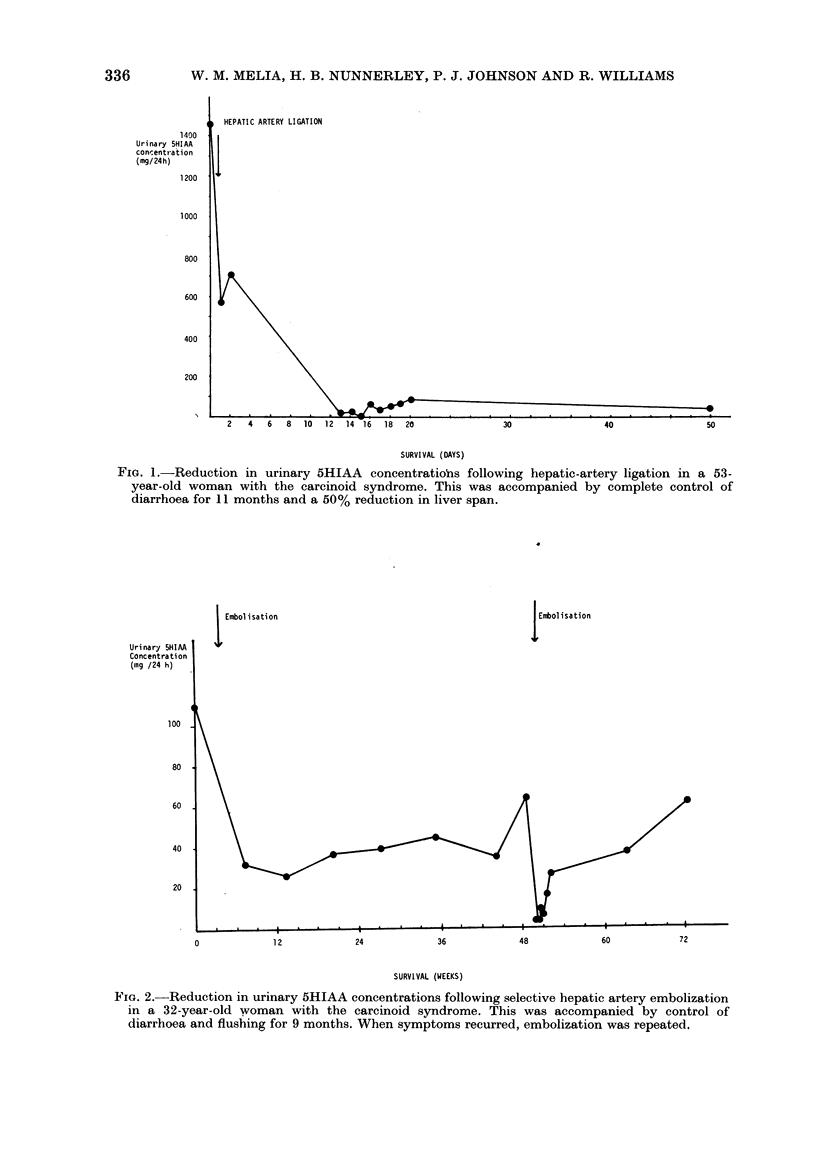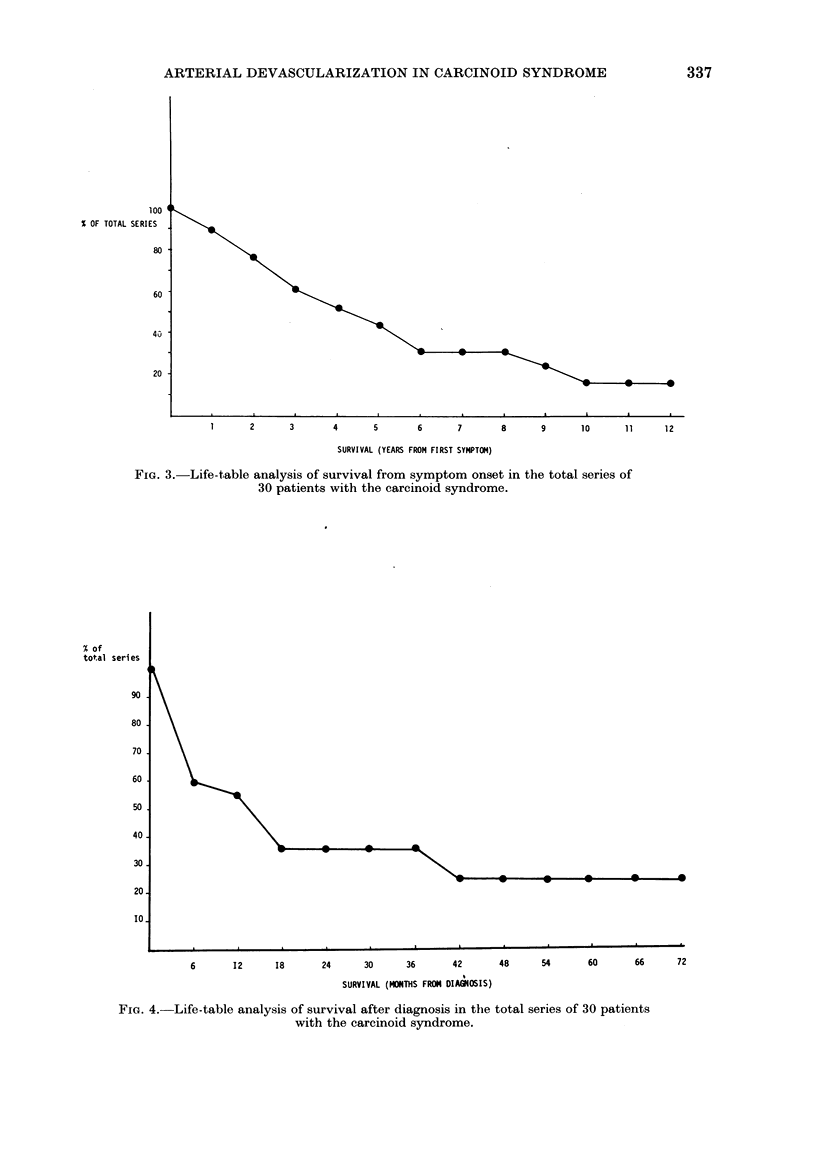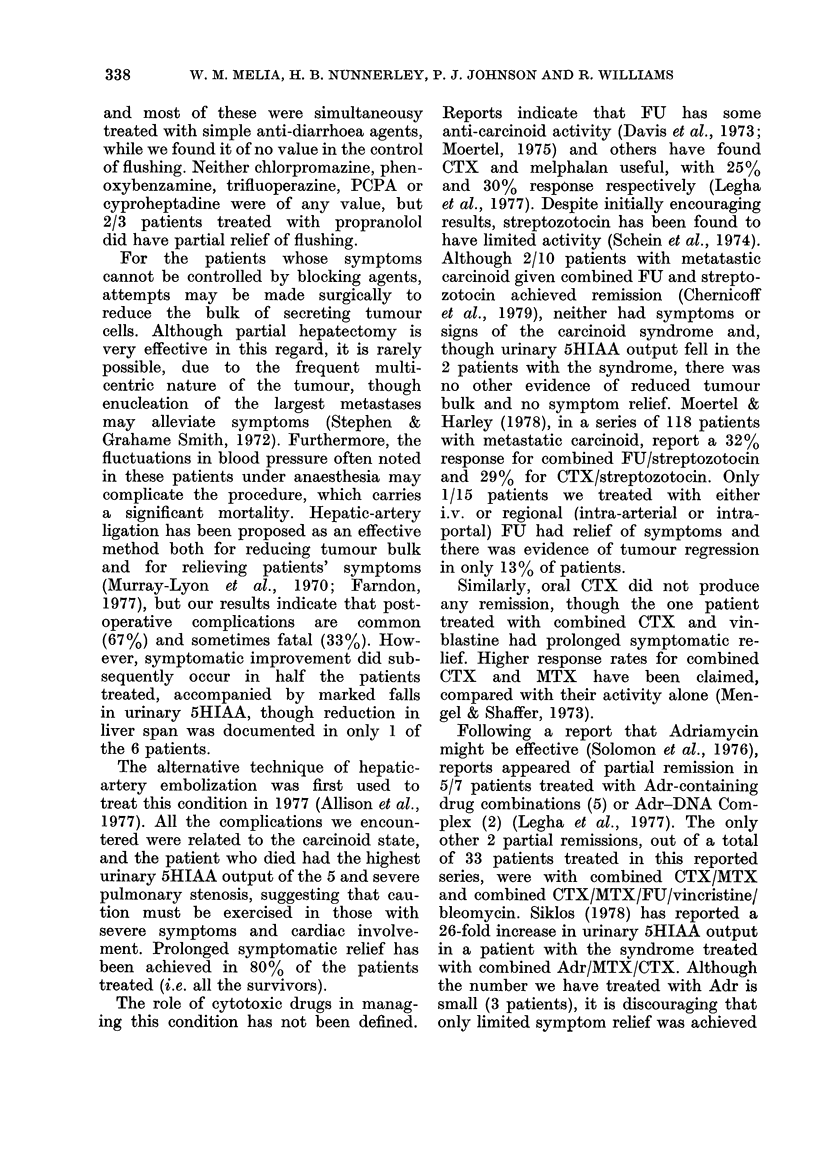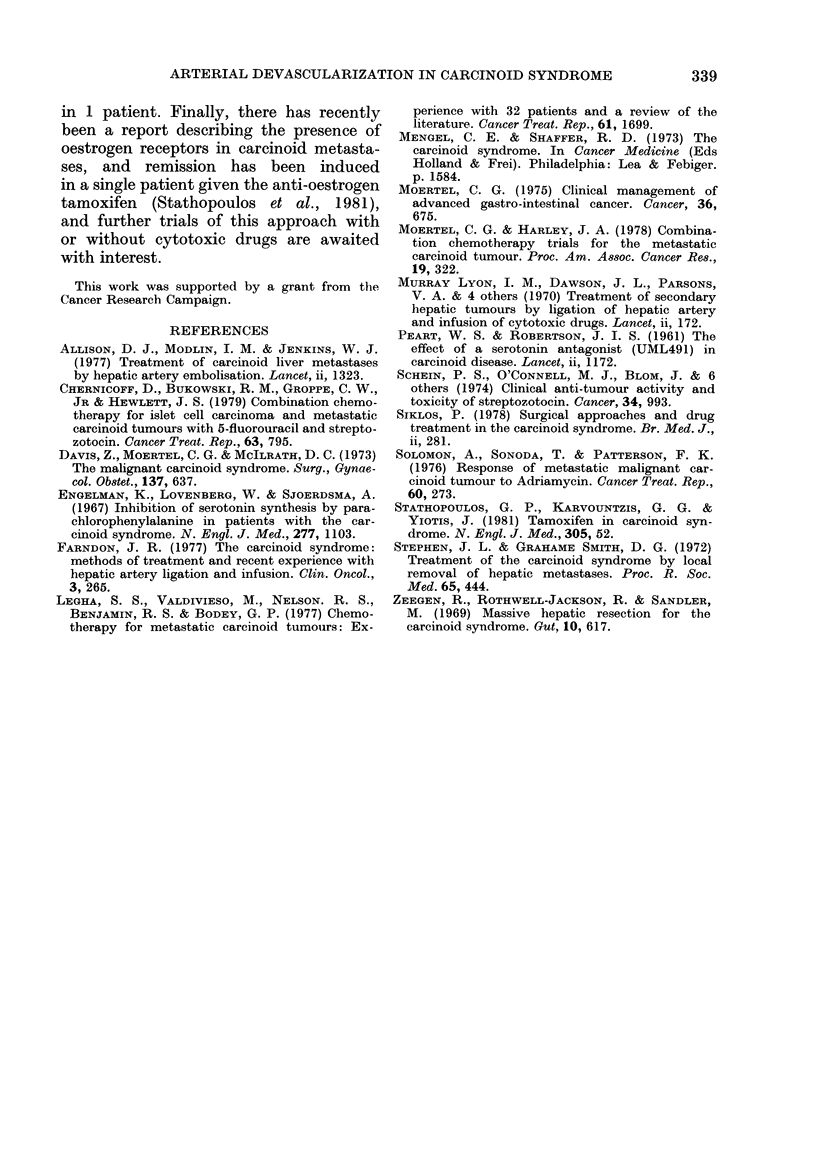# Use of arterial devascularization and cytotoxic drugs in 30 patients with the carcinoid syndrome.

**DOI:** 10.1038/bjc.1982.208

**Published:** 1982-09

**Authors:** W. M. Melia, H. B. Nunnerley, P. J. Johnson, R. Williams

## Abstract

Thirty patients with symptoms of the carcinoid syndrome and other symptoms not controlled by pharmacological agents were analysed with respect to the value of various treatment measures used. Tumour devascularization was carried out in 11 patients, either by surgical ligation of the main hepatic artery (6) or by percutaneous arterial embolization (5). The latter was shown to be the safer technique, both with respect to initial morbidity/mortality and other side effects. Control of flushing and diarrhoea was achieved in 80% and the technique was also repeated on one occasion with success when symptoms recurred. The use of cytotoxic drugs alone, including 5-fluorouracil, cyclophosphamide and Adriamycin produced symptomatic relief in only 4 of the 22 patients treated. They should only be considered if devascularization by arterial embolization proves impossible or cannot be repeated when symptoms recur.


*Br. J. Cancer* (1982) **46**, 331

### USE OF ARTERIAL DEVASCULARIZATION AND CYTOTOXIC DRUGS IN 30 PATIENTS WITH THE CARCINOID SYNDROME

W. M. MELIA, H. B. NUNNERLEY, P. J. JOHNSON AND R. WILLIAMS

*From the Liver Unit and the Department of Diagnostic Radiology, King's College Hospital and Medical School, Denmark Hill, London SE5*

Received 1 March 1982 Accepted 21 April 1982

**Summary.—Thirty patients with symptoms of the carcinoid syndrome and other symptoms not controlled by pharmacological agents were analysed with respect to the value of various treatment measures used. Tumour devascularization was carried out in 11 patients, either by surgical ligation of the main hepatic artery (6) or by percutaneous arterial embolization (5). The latter was shown to be the safer technique, both with respect to initial morbidity/mortality and other side effects. Control of flushing and diarrhoea was achieved in 80% and the technique was also repeated on one occasion with success when symptoms recurred. The use of cytotoxic drugs alone, including 5-fluorouracil, cyclophosphamide and Adriamycin produced symptomatic relief in only 4 of the 22 patients treated. They should only be considered if devascularization by arterial embolization proves impossible or cannot be repeated when symptoms recur.**

ALTHOUGH THE PRESENCE of the carcinoid syndrome almost invariably implies hepatic metastases, some of the tumours continue to grow only slowly and patients may live for many years. Various pharmacological agents can be helpful in the control of the diarrhoea and flushing, including methysergide, a 5-hydroxytryptamine (5-HT) antagonist (Peart & Robertson, 1961), parachlorophenylalanine (PCPA), an inhibitor of 5-HT synthesis (Engelman *et al.*, 1967) and cyproheptadine, a 5-HT antagonist. Chlorpromazine, by its α-adrenergic blocking effect and by allaying stressful situations which frequently provoke symptoms (Farndon, 1977), and propranolol may give partial relief of flushing (Zeegen *et al.*, 1969).

However, in some patients the condition is resistant, and the alternative approach is a direct attack to reduce the size of functioning tumour mass on which the severity of symptoms depends, by using cytotoxic drugs. 5-Fluorouracil (Davis *et al.*, 1973; Moertel, 1975), cyclophosphamide, melphalan (Legha *et al.*, 1977), streptozotocin (Schein, *et al.*, 1974) and Adriamycin (Solomon *et al.*, 1976; Legha *et al.*, 1977) have been shown to be of some value, but appear to be much less effective in patients with the carcinoid syndrome than in those with asymptomatic, possibly more malignant, metastatic carcinoid (Legha *et al.*, 1977).

In the present report we have reviewed our experience with 30 patients referred over the last 10 years with symptoms of the carcinoid syndrome, or other symptoms such as severe pain not controllable by pharmacological agents, in whom various chemotherapeutic schedules and hepatic-devascularization techniques have been used. Specific monitoring with urinary 5-hydroxyindoleacetic acid (5HIAA) concentrations has allowed objective assessment of the responses.

### PATIENTS AND METHODS

Thirty patients were referred to the Liver Unit between 1969 and 1979. Sixteen were male and 14 female, with a median age at diagnosis of 56·5 years (range 24–76). The site of the primary tumour was ascertained in 22 patients as ileum (13), colon (3), bronchus (2), pancreas (2), caecum (1) and rectum (1). Primary resection had been performed in 14 patients, mainly on account of the development of intestinal obstruction. The hepatic tumour deposits appeared to be multiple on the basis of the vascular pattern seen on selective hepatic angiography in 14/21 patients, whilst the remainder appeared to have solitary lesions.

Symptoms leading to referral were uncontrolled diarrhoea (28 patients) and flushing (25). In 2 patients, the major symptom was severe right subcostal pain with weight loss. Cardiac involvement was detected in 16 patients, 10 of whom had pulmonary stenosis and 7 tricuspid incompetence. Methysergide had been used to control diarrhoea with partial success in 10/15 patients treated, 3 of whom also received codeine phosphate. One of 3 patients treated with indomethacin had shown temporary improvement of diarrhoea. Eight patients had been treated with cyproheptadine, with no appreciable success, whilst partial relief of flushing had been achieved in 2/3 patients given propranolol. A variety of other agents—flufenamic acid (3), parachlorophenylalanine (1), phenoxybenzamine (1), chlorpromazine (1) and trifluoperazine (1)—had been used, without obvious benefit.

*Devascularization and cytotoxic regimens.*—Devascularization procedures were performed in 11 patients, by ligation of the main hepatic artery at laparotomy in 5, by selective ligation of the right hepatic artery supplying the main tumour mass in 1, and by percutaneous arterial embolization (usually at the time of initial arteriographic examination) in the remainder. In 1 patient, embolization was repeated on one occasion. Embolization was performed by injecting chopped-up gel foam mixed with contrast medium into selectively catheterized branches of the hepatic artery supplying tumour, under general anaesthesia. Because of the danger of release of vasoactive agents as a result of the necrosis induced, patients were prophylactically given blocking agents for 4 days beforehand: PCPA 500 mg q.d.s., cyproheptadine 4 mg q.d.s. and methysergide 3 mg q.d.s. Prophylactic antibiotics were given: benzyl-pencillin 1 mega unit q.d.s., gentamicin 80 mg t.d.s. and metronidazole 500 mg q.d.s. i.v.—for at least 7 days after embolization.

Cytotoxic drugs used included 5-fluorouracil (FU, 15 patients), cyclophosphamide (CTX, 6), and Adriamycin (Adr, 3). FU was given by intra-arterial infusion in 12 patients for periods of 1–14 days, total doses being 0·3–18·4 g. In an attempt to reduce the risk of release of vasoactive agents, this technique was used as a preliminary step before ligation in 5, while it was used subsequent to ligation in 1 of these and in 1 other patient. In 3 patients, FU was given by portal-vein infusion, whilst 8 were treated with i.v. FU. CTX was administered orally to 6 patients in doses of 50–100 mg/day. Three were treated with i.v. Adr in a dose of 60 mg/m$^2$. One patient, in whom i.v. FU appeared to have no effect, was given a combination of oral CTX (600 mg), i.v. MTX (40 mg), vincristine (2 mg) and FU (2·5 g), while another was given combined CTX and vinblastine for 4 years.

### RESULTS

### *Devascularization procedures*

Reduction in frequency and severity of diarrhoea and flushing occurred in 3/6 patients treated by hepatic-artery ligation, in 2 of whom improvement was temporarily complete (11 and 65 months, respectively) (Table). This was accompanied by a marked reduction in urinary 5-hydroxyindoleacetic acid (5HIAA) concentration (Fig. 1) which was recorded in 1 other patient without symptom relief (mean fall: $94 \pm 8\%$). Reduction in liver size appeared to occur in only 1 patient, in whom a 50% reduction in liver span in the right mid-clavicular line was clinically detected. Postoperative complications (fluctuations in blood pressure (2), peritonitis (1), septicaemia (1), hallucinations (1), marked exacerbation of flushing lasting 3 days (1)) arose in 4/6 patients, 2 of whom died 3 and 6 days later respectively.

After arterial embolization 4/5 patients noted symptomatic improvement, and this was accompanied by a fall in urinary 5HIAA excretion (mean: $79 \pm 15\%$; Fig.

2). The procedure was followed by fluctuations in blood pressure in 2 patients, delayed awakening with a prolonged confusional state in 2, and 1 woman with severe pulmonary stenosis and the highest urinary 5HIAA concentrations seen (1032 mg/24 h) died within a week of embolization from right-sided cardiac failure.

Including both types of devascularization techniques together, symptomatic relief resulted in 7/11 patients treated. The mean duration of symptomatic remission was 13·5 months (range 7–65), and remission lasted >1 year in 4 and >2 years in 1 patient.

*Cytotoxic drugs*

Of the 15 patients given FU, complete control of flushing and diarrhoea lasting 4·5 months was achieved in 1 patient, in whom there was no evidence of reduction in liver size. A 50% reduction in liver span in the right midclavicular line was detected clinically in 1 other patient, who did not have a symptomatic response, and there was some reduction in urinary 5HIAA output in 2 others (33% and 70% respectively). Complications followed the use of intra-arterial FU in 10/12 patients thus treated, with septicaemia in 5 patients (2 of whom died), catheter obstruction in 2 and common iliac artery thrombotic obstruction consequent on catheter displacement in 1. No complications resulted from intraportal FU infusion (3 patients) but 4/8 patients given i.v. FU developed complications (septicaemia 3, dysgeusia 1) and 2 died from sepsis.

One of 3 patients treated with Adr experienced partial relief of diarrhoea and pain lasting 6 months, and no patients suffered side-effects. There was no evidence of drug response in any of the 6 patients given CTX, none of whom suffered side-effects. The single patient treated with combined CTX/MTX/vincristine/FU died within 1 week from sepsis complicating myelosuppression, but the patient given combined CTX and vinblastine had prolonged symptom relief (9 months).

*Survival analysis in relation to treatment schedules*

When survival of the total series of patients from the onset of symptoms of the carcinoid syndrome was determined by the life-table method (Fig. 3), 1- and 2-year survival rates of 90% and 76% respectively were obtained. Five-year survival was 44% and 10-year survival 16%. Mean symptom duration before diagnosis was 30 months. When survival was measured from time of diagnosis (Fig. 4) 1- and 2-year survival rates of 58% and 38% respectively were obtained, whilst the 5-year survival was 23%.

The overall survival from diagnosis of those patients treated by hepatic-arterial ligation was similar to survival of the total series: 50% 1-year and 17% 2-year survival. After arterial embolization, the corresponding figures were 80% and 80% and, with the exception of the patient dying within a week of the procedure, the others are still alive at 19, 24, 24, and and 26 months respectively. For the series of 15 patients treated with FU, 1-year survival was 37% and 2-year, 15%.

### DISCUSSION

Although our series of patients is a highly selected one, because they had refractory symptoms referred to a specialist unit, it is interesting that, when measured from the first related symptom, the survival figures obtained are very similar to those reported by Davis *et al.* (1973), who found that median life-span from the first episode of flushing in 60 patients was 38 months, with a 6-year survival rate of 25%. Despite this relatively good prognosis, therapeutic intervention is frequently indicated, both to control troublesome symptoms and also to induce tumour regression, as only 58% of the patients in this series survived 1 year from the time of diagnosis.

Of the various pharmacological agents used to control diarrhoea and flushing, methysergide helped control diarrhoea in only one-third of those we treated,

TABLE.—*Details of 11 patients treated by devascularization*

| No. | Sex/age | Symptom duration (mths) | Survival (mths) Ex symptoms | Ex devasc. | Urinary 5HIAA at presentation (mg/24 h) | %age fall | Duration of symptomatic response (mths) | Blocking agents | Antibiotic cover | Complications | Cytotoxic drug treatment |
|---|---|---|---|---|---|---|---|---|---|---|---|
| 1 | M/79 | 4·5 | 17·5 | 13·0 | 118 | 91·5 | 7 | — | — | — | Intra-arterial FU × 11 days pre-HAL, intra-portal FU × 32 days post-HAL |
| 2 | F/44 | 10 | 10 | — | 180 | 100 | — | Cyproheptadine methysergide | — | Hallucinations, anaemia, elevated transaminases, abdominal pain, hypo-tension, died 6 days later | Intra-arterial FU × 7 days 11 days pre-HAL |
| 3 | F/44 | 63 | 63 | — | 230 | — | — | — | — | Cardiac arrhyth-mias, fluctuat-ing blood pressure, septicaemia died 3 days later | — |
| 4 | M/51 | 14 | 98 | 84 | — | — | 65 | Trasylol | Penicillin G | Pleuritic pain | Intra-arterial FU pre-HAL and × 2 months post-HAL |

| | | | | | | | | | | | |
|---|---|---|---|---|---|---|---|---|---|---|---|
| 5 | F/53 | 25 | 30 | 5 | 1416 | 99 | 11 | Cyproheptadine methysergide | — | Vasodilatation, peritonitis, pleuritic pain, alopecia | Intra-portal FU × 4 days post-HAL |
| 6 | M/46 | 51 | 51·75 | 0·75 | 49 | 83·7 | — | Methysergide flufenamic acid | — | Died 24 days later from complications of intra-arterial cytotoxic drug treatment | Intra-portal FU × 4 days post-HAL |
| 7 | F/62 | 84 | 84 | — | 1032 | — | — | Methysergide + cyproheptadine + PCPA | Penicillin G + gentamicin + metronidazole | Fluctuating blood pressure, delayed awakening from anaesthetic, pneumonia, right heart failure, died 11 days later | — |
| 8 | F/72 | 132 | 150 | 18 | 75 | 66 | 18 | ,, | ,, | Hypertension | — |
| 9 | F/70 | 24 | 49 | 25 | 107 | 77 | 21 | ,, | ,, | Hypotension | — |
| 10 | M/67 | 84 | 106 | 22 | 793 | 100 | 16 | ,, | ,, | Delayed awakening from anaesthetic, confusion (2/52) | — |
| 11 | F/32 | 97 | 120 | (A) 23<br>(B) 11 | 110 | (A) 71<br>(B) — | (A) 9<br>(B) 7 | ,,<br>,, | ,,<br>,, | (A) Hypotension<br>(B) — | — |

FIG. 1.—Reduction in urinary 5HIAA concentrations following hepatic-artery ligation in a 53-year-old woman with the carcinoid syndrome. This was accompanied by complete control of diarrhoea for 11 months and a 50% reduction in liver span.

FIG. 2.—Reduction in urinary 5HIAA concentrations following selective hepatic artery embolization in a 32-year-old woman with the carcinoid syndrome. This was accompanied by control of diarrhoea and flushing for 9 months. When symptoms recurred, embolization was repeated.

FIG. 3.—Life-table analysis of survival from symptom onset in the total series of 30 patients with the carcinoid syndrome.

FIG. 4.—Life-table analysis of survival after diagnosis in the total series of 30 patients with the carcinoid syndrome.

and most of these were simultaneousy treated with simple anti-diarrhoea agents, while we found it of no value in the control of flushing. Neither chlorpromazine, phenoxybenzamine, trifluoperazine, PCPA or cyproheptadine were of any value, but 2/3 patients treated with propranolol did have partial relief of flushing.

For the patients whose symptoms cannot be controlled by blocking agents, attempts may be made surgically to reduce the bulk of secreting tumour cells. Although partial hepatectomy is very effective in this regard, it is rarely possible, due to the frequent multicentric nature of the tumour, though enucleation of the largest metastases may alleviate symptoms (Stephen & Grahame Smith, 1972). Furthermore, the fluctuations in blood pressure often noted in these patients under anaesthesia may complicate the procedure, which carries a significant mortality. Hepatic-artery ligation has been proposed as an effective method both for reducing tumour bulk and for relieving patients' symptoms (Murray-Lyon *et al.*, 1970; Farndon, 1977), but our results indicate that postoperative complications are common (67%) and sometimes fatal (33%). However, symptomatic improvement did subsequently occur in half the patients treated, accompanied by marked falls in urinary 5HIAA, though reduction in liver span was documented in only 1 of the 6 patients.

The alternative technique of hepatic-artery embolization was first used to treat this condition in 1977 (Allison *et al.*, 1977). All the complications we encountered were related to the carcinoid state, and the patient who died had the highest urinary 5HIAA output of the 5 and severe pulmonary stenosis, suggesting that caution must be exercised in those with severe symptoms and cardiac involvement. Prolonged symptomatic relief has been achieved in 80% of the patients treated (*i.e.* all the survivors).

The role of cytotoxic drugs in managing this condition has not been defined. Reports indicate that FU has some anti-carcinoid activity (Davis *et al.*, 1973; Moertel, 1975) and others have found CTX and melphalan useful, with 25% and 30% response respectively (Legha *et al.*, 1977). Despite initially encouraging results, streptozotocin has been found to have limited activity (Schein *et al.*, 1974). Although 2/10 patients with metatastic carcinoid given combined FU and streptozotocin achieved remission (Chernicoff *et al.*, 1979), neither had symptoms or signs of the carcinoid syndrome and, though urinary 5HIAA output fell in the 2 patients with the syndrome, there was no other evidence of reduced tumour bulk and no symptom relief. Moertel & Harley (1978), in a series of 118 patients with metastatic carcinoid, report a 32% response for combined FU/streptozotocin and 29% for CTX/streptozotocin. Only 1/15 patients we treated with either i.v. or regional (intra-arterial or intraportal) FU had relief of symptoms and there was evidence of tumour regression in only 13% of patients.

Similarly, oral CTX did not produce any remission, though the one patient treated with combined CTX and vinblastine had prolonged symptomatic relief. Higher response rates for combined CTX and MTX have been claimed, compared with their activity alone (Mengel & Shaffer, 1973).

Following a report that Adriamycin might be effective (Solomon *et al.*, 1976), reports appeared of partial remission in 5/7 patients treated with Adr-containing drug combinations (5) or Adr–DNA Complex (2) (Legha *et al.*, 1977). The only other 2 partial remissions, out of a total of 33 patients treated in this reported series, were with combined CTX/MTX and combined CTX/MTX/FU/vincristine/bleomycin. Siklos (1978) has reported a 26-fold increase in urinary 5HIAA output in a patient with the syndrome treated with combined Adr/MTX/CTX. Although the number we have treated with Adr is small (3 patients), it is discouraging that only limited symptom relief was achieved in 1 patient. Finally, there has recently been a report describing the presence of oestrogen receptors in carcinoid metastases, and remission has been induced in a single patient given the anti-oestrogen tamoxifen (Stathopoulos *et al.*, 1981), and further trials of this approach with or without cytotoxic drugs are awaited with interest.

This work was supported by a grant from the Cancer Research Campaign.

### REFERENCES

ALLISON, D. J., MODLIN, I. M. & JENKINS, W. J. (1977) Treatment of carcinoid liver metastases by hepatic artery embolisation. *Lancet*, ii, 1323.

CHERNICOFF, D., BUKOWSKI, R. M., GROPPE, C. W., JR & HEWLETT, J. S. (1979) Combination chemotherapy for islet cell carcinoma and metastatic carcinoid tumours with 5-fluorouracil and streptozotocin. *Cancer Treat. Rep.*, **63**, 795.

DAVIS, Z., MOERTEL, C. G. & MCILRATH, D. C. (1973) The malignant carcinoid syndrome. *Surg., Gynaecol. Obstet.*, **137**, 637.

ENGELMAN, K., LOVENBERG, W. & SJOERDSMA, A. (1967) Inhibition of serotonin synthesis by parachlorophenylalanine in patients with the carcinoid syndrome. *N. Engl. J. Med.*, **277**, 1103.

FARNDON, J. R. (1977) The carcinoid syndrome: methods of treatment and recent experience with hepatic artery ligation and infusion. *Clin. Oncol.*, **3**, 265.

LEGHA, S. S., VALDIVIESO, M., NELSON. R. S., BENJAMIN, R. S. & BODEY, G. P. (1977) Chemotherapy for metastatic carcinoid tumours: Experience with 32 patients and a review of the literature. *Cancer Treat. Rep.*, **61**, 1699.

MENGEL, C. E. & SHAFFER, R. D. (1973) The carcinoid syndrome. In *Cancer Medicine* (Eds Holland & Frei). Philadelphia: Lea & Febiger. p. 1584.

MOERTEL, C. G. (1975) Clinical management of advanced gastro-intestinal cancer. *Cancer*, **36**, 675.

MOERTEL, C. G. & HARLEY, J. A. (1978) Combination chemotherapy trials for the metastatic carcinoid tumour. *Proc. Am. Assoc. Cancer Res.*, **19**, 322.

MURRAY LYON, I. M., DAWSON, J. L., PARSONS, V. A. & 4 others (1970) Treatment of secondary hepatic tumours by ligation of hepatic artery and infusion of cytotoxic drugs. *Lancet*, ii, 172.

PEART, W. S. & ROBERTSON, J. I. S. (1961) The effect of a serotonin antagonist (UML491) in carcinoid disease. *Lancet*, ii, 1172.

SCHEIN, P. S., O'CONNELL, M. J., BLOM, J. & 6 others (1974) Clinical anti-tumour activity and toxicity of streptozotocin. *Cancer*, **34**, 993.

SIKLOS, P. (1978) Surgical approaches and drug treatment in the carcinoid syndrome. *Br. Med. J.*, ii, 281.

SOLOMON, A., SONODA, T. & PATTERSON, F. K. (1976) Response of metastatic malignant carcinoid tumour to Adriamycin. *Cancer Treat. Rep.*, **60**, 273.

STATHOPOULOS, G. P., KARVOUNTZIS, G. G. & YIOTIS, J. (1981) Tamoxifen in carcinoid syndrome. *N. Engl. J. Med.*, **305**, 52.

STEPHEN, J. L. & GRAHAME SMITH, D. G. (1972) Treatment of the carcinoid syndrome by local removal of hepatic metastases. *Proc. R. Soc. Med.* **65**, 444.

ZEEGEN, R., ROTHWELL-JACKSON, R. & SANDLER, M. (1969) Massive hepatic resection for the carcinoid syndrome. *Gut*, **10**, 617.